# Salvage High-Dose-Rate Interventional Radiotherapy (Brachytherapy) Combined with Surgery for Regionally Relapsed Head and Neck Cancers

**DOI:** 10.3390/cancers15184549

**Published:** 2023-09-14

**Authors:** Tamer Soror, Justina Paul, Corinna Melchert, Christian Idel, Dirk Rades, Karl-Ludwig Bruchhage, György Kovács, Anke Leichtle

**Affiliations:** 1 Radiation Oncology Department, University of Lübeck/UKSH-CL, 23562 Lübeck, Germany; corinna.melchert@uksh.de (C.M.); dirk.rades@uksh.de (D.R.); 2 National Cancer Institute (NCI), Radiation Oncology Department, Cairo University, Giza 12613, Egypt; 3 Department of Oto-Rhino-Laryngology and Head and Neck Surgery, University of Lübeck, 23562 Lübeck, Germany; justina.paul@yahoo.de (J.P.); christian.idel@uksh.de (C.I.); karl-ludwig.bruchhage@uksh.de (K.-L.B.); anke.leichtle@uksh.de (A.L.); 4 Università Cattolica del Sacro Cuore, Gemelli-INTERACTS, 00168 Rome, Italy; kovacsluebeck@gmail.com

**Keywords:** interventional radiotherapy, high-dose-rate brachytherapy, head and neck cancer, local recurrence

## Abstract

**Simple Summary:**

Many head and neck cancer patients suffer from recurrence of the tumor after previous treatment. The initial treatment usually entails operation, radiation treatment, chemotherapy, or combinations of these treatments. For those patients who have no distant metastases, there are limited options of curative treatments. Usually, surgery would be the treatment of choice. However, not all patients are operable, and the treatment results are not satisfactory. Interventional high-dose-rate radiotherapy (HDR-IRT, brachytherapy) is a special method of radiotherapy with unique physical properties. Re-irradiation with HDR-IRT combined with surgery improves the outcome and offers the patients a second chance of curative treatment. After three years of the treatment, 88% of the patients had no local recurrences. A minority of patients, approximately 35%, experienced significant yet controllable complications throughout the follow-up duration. We have confidence that this perioperative and multidisciplinary approach yields favorable clinical results with a tolerable incidence of side effects.

**Abstract:**

(1) Background: to report on the use of high-dose-rate (HDR) interventional radiotherapy (brachytherapy, IRT) as a salvage treatment for patients with regionally relapsed head and neck cancers. (2) Methods: A retrospective study of 60 patients treated with HDR-IRT for loco-regionally relapsed head and neck cancers at our institution (2016–2020). Treatment procedure, results, and related toxicities were collected. Local and overall survival outcomes were analyzed. (3) Results: The median follow-up was 22.4 months. Twenty-nine (48.3%) patients had locoregional recurrences with a median time of 28.9 months. The local-recurrence free-survival was 88.1% and 37.3% at 3 years and 5 years. At the last follow-up, 21 patients were alive and the median time to death was 24 months. The overall survival was 39.2% and 16.6% at 3 years and 5 years. Collectively, there were 28 events of grade ≥ 3 late toxicities recorded in 21 patients (35%). (4) Conclusions: Salvage HDR-IRT combined with surgery offers a second-line curative treatment option for regionally relapsed head and neck cancers with acceptable outcomes and toxicities.

## 1. Introduction

The management of locoregional recurrences of head and neck cancers after initial treatment is still a major challenge for clinicians worldwide [1,2]. The rates of locoregional recurrences vary according to tumor location, stage, histology parameters, and applied primary treatment, ranging between 15–50% [1,2,3,4,5]. The recurrences usually have aggressive features such as undifferentiated grade, perineural invasion, lymphovascular invasion, extracapsular spread, and extensive soft tissue infiltration [2,6,7]. The salvage treatment options are highly dependent on the used treatment modalities in primary management. Salvage radical surgical resection is only possible in less than 50% of the patients and carries a high potential risk of postoperative complications as well as functional and cosmetic long-term sequelae. The risk of local failure is as high as 59% [1,8].

For patients who are not considered candidates for curative surgery, re-irradiation with or without chemotherapy would be a potentially curative treatment [1,9,10,11]. However, the toxicity profile of re-irradiation using external beam radiation treatment (EBRT) is relatively high with modest outcomes [10,11,12]. The introduction of modern EBRT techniques such as intensity-modulated radiotherapy (IMRT) and stereotactic body radiotherapy (SBRT), as well as the use of proton-beam radiotherapy and other hadrontherapies, had slightly improved the toxicity rates [9,13,14,15,16,17,18,19,20,21,22].

High-dose-rate interventional radiotherapy (HDR-IRT, brachytherapy) represents a remarkably precise and conformal irradiation technique that facilitates significant dose escalation through the application of extreme hypofractionation. The implementation of this approach leads to a steep dose gradient, ensuring the protection of vital organs at risk (OAR). Consequently, HDR-IRT emerges as a viable and effective salvage modality for managing-recurrent cancers in different anatomical locations [23,24,25,26,27,28,29].

At our institute, we adapted an interdisciplinary salvage approach that combines surgery, whether radical or debulking, with HDR-IRT for locally or locoregionally recurrent head and neck cancers. The present study reports on the oncological results and the treatment-related toxicities of this approach.

## 2. Materials and Methods

### 2.1. Patients

We conducted a comprehensive review of the medical records for head and neck cancer patients who underwent salvage HDR-IRT with surgery for histologically confirmed recurrences between January 2016 and December 2020. All patients were evaluated through a physical examination, a pan-endoscopy, an ultrasound examination of the neck, and laboratory investigations. Radiographic and/or isotopic scans were performed before treatment. The individualized treatment decision for each patient was taken throughout a multidisciplinary tumor board for head and neck cancers. A signed informed consent was collected from all patients prior to treatment.

### 2.2. Interventional Radiotherapy (Brachytherapy)

**Figure 1:**
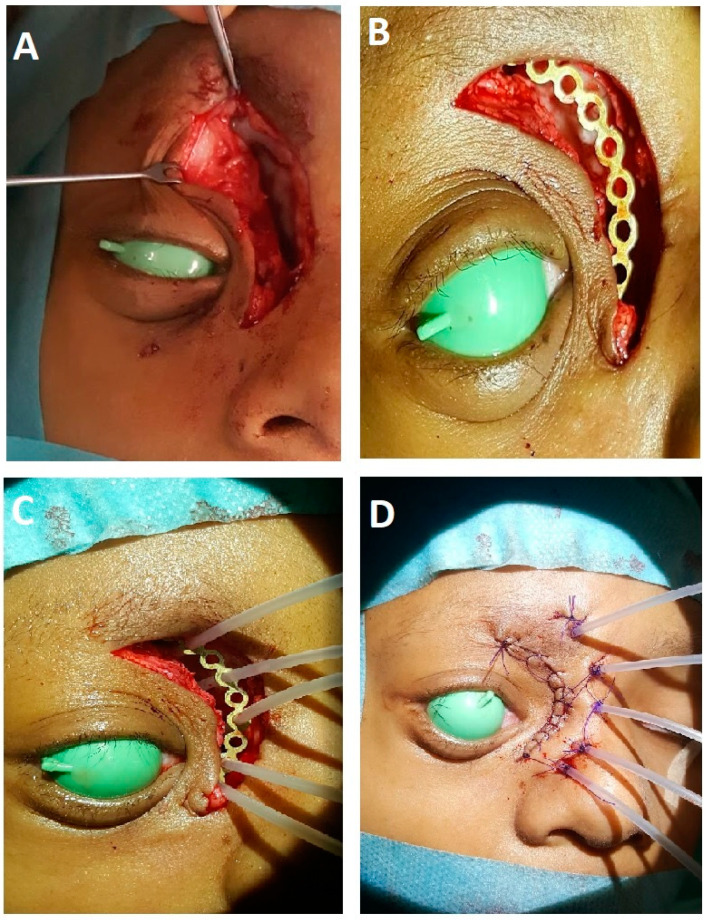
Recurrent tumor of medial orbital wall. (**A**): after tumor debulking; (**B**): insertion of fixation metal plate; (**C**): implantation of the interstitial catheters; (**D**): after closure of wound and suturing of catheters.

During the operation, the interventional radiotherapy expert together with the surgeon jointly assessed the anatomical context, identifying any potential sites of residual tumor, whether on a microscopic or macroscopic scale. Interstitial plastic catheters were then implanted in a parallel fashion, maintaining a spacing of 8–12 mm between them, covering the target region and incorporating safety margins of 15–20 mm around the tumor bed, as illustrated in Figure 1. If indicated, soft tissue reconstruction with a pedicled or a free flap was performed first, then the interstitial catheters were implanted before the final suture.

**Figure 2:**
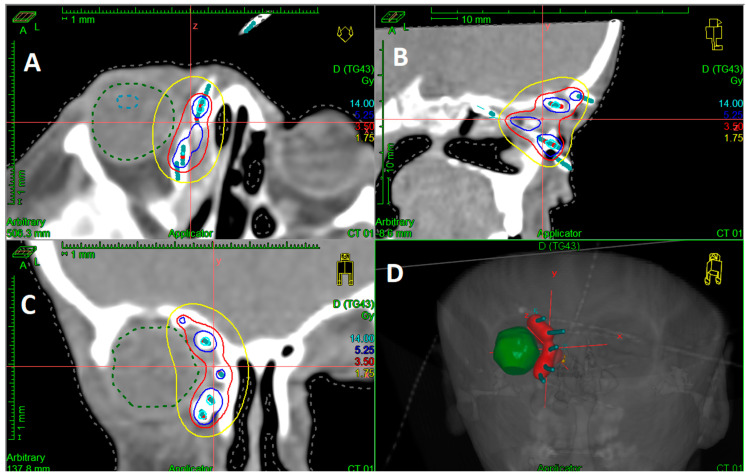
Dose distribution; (**A**): transversal, (**B**): sagittal; (**C**): coronal; (**D**): three−dimensional reconstruction.

On the second or third day following the surgery, a thin-slice simulation CT scan was conducted and then imported into the treatment planning system (TPS). The three-dimensionally reconstructed CT images were used for catheter reconstruction, delineation of clinical target volume (CTV), and organs at risk (OAR). Dose planning/optimization were performed either as forward planning or recently using inverse-planning tools of the TPS (Oncentra planning; Elekta Brachytherapy, Veenendaal, The Netherlands). CTV included the tumor bed with safety margins (15–20 mm) and excluded the skin unless infiltrated. HDR-IRT started within a timeframe of 2–5 days post-surgery, depending on the type of surgical procedure and the patient’s overall condition. The prescribed dose was delivered over approximately 5 days, with fractions delivered twice daily, maintaining a minimum interval of 6 h between each fraction. This fractionation schedule was chosen as it has a higher radiobiological effectiveness. A sample of the three-dimension dose distribution is shown in Figure 2.

### 2.3. Follow-Up

Follow-up appointments were initially set at three-month intervals for a duration of three years, after which they were scheduled every six months. Pan-endoscopy with imaging investigations were performed 1, 2, and 5 years posttreatment of an oncological certified standard follow-up procedure, and otherwise only if needed. The assessment of treatment-related toxicities was carried out based on the Common Terminology Criteria for Adverse Events 5.0 grading system.

### 2.4. Statistical Analysis

The results were presented both as an absolute value and as a median along with their corresponding range. Probability estimates of recurrence-free survival (RFS) and overall survival (OS) were calculated through the Kaplan–Meier analysis method. Statistical analyses were conducted using SPSS-V.20. (IBM-Corp., New York, NY, USA).

### 2.5. Ethics Committee/Informed Consent

This study was approved by the human subjects ethics board of the University of Luebeck and was conducted in accordance with the Helsinki Declaration of 1975, as revised in 2013. (Acceptance number: 20-489).

## 3. Results

### 3.1. Patient and Tumor Characteristics

We identified 60 patients who were treated with surgery and HDR-IRT for regionally relapsed head and neck cancer. Patients and disease characteristics are summarized in Table 1. The median age at HDR-IRT was 65.6 years (range: 35.5–92.7). Most patients were relapsed locally at the primary tumor site (68.3%); regional LN recurrences occurred in 23.3%.

### 3.2. Initial Treatments

The initial treatments of primary tumors are described in Table 1. All patients were initially operated with 33.3% of the patients having received a primary neck dissection. Forty-two (70%) patients had received EBRT, and 27 (45%) patients had received chemotherapy.

### 3.3. Salvage Treatment

All patients were operated on with implantation of the interstitial HDR-IRT catheters. In nine (15%) patients, an organ preserving surgery was performed. Twenty-seven (45%) patients had only a debulking operation (gross residual) due to the anatomical situation, such as infiltration into the internal carotid artery; in eleven (18.3%) patients the surgical margins were invaded with tumor cells (microscopic residual); meanwhile, four (5%) patients had close surgical margin (<5 mm).

### 3.4. HDR-IRT Treatment Characteristics

In the majority of patients (88.3%), HDR-IRT was the only salvage irradiation technique. The median dose of HDR-IRT for all patients was 30 Gy, delivered in two daily fractions. The median fraction dose was 3 Gy, Table 2.

In seven (11.7%) patients, HDR-IRT was combined with EBRT. Four patients were previously irradiated with 30–50 Gy initially and re-irradiated with 30–50 Gy and 12–20 Gy HDR-IRT. Three patients had no previous EBRT, and they were treated with 30–50 Gy EBRT and 30 Gy HDR-IRT, Table 2.

### 3.5. Treatment Outcome

**Figure 3:**
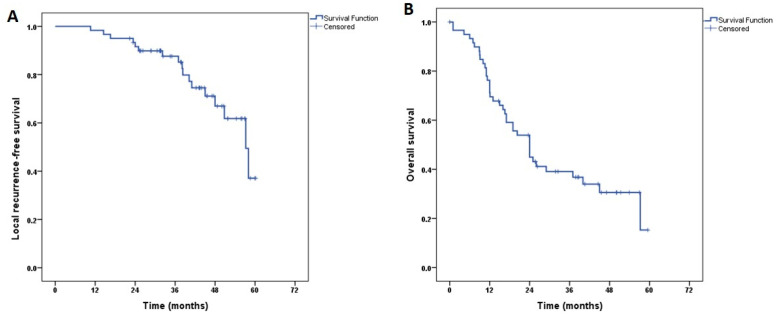
(**A**): Recurrence-free survival; (**B**): Overall survival.

The median duration of the follow-up period was 22.4 months (range: 3.6–63.5). Twenty-nine (48.3%) patients had locoregional recurrences, fifteen (25%) patients relapsed locally at the treated region, and fourteen (23.3%) patients developed regional nodal relapse. The median time to develop LR following HDR-IRT was 28.9 months (3–56.9). The RFS was 88.1% and 37.3% at 3 years and 5 years, as shown in Figure 3.

At the last follow-up, 21 patients were alive and the median time to death was 24 months (4.3–59.5). The OS was 39.2% and 16.6% at 3 years and 5 years, as shown in Figure 3.

### 3.6. Treatment-Related Toxicities

The encountered acute and late treatment-related toxicities are detailed in Table 3. The most common grade ≥ 3 acute toxicity was post-operative pain (16.7%), which was controlled in all patients using a patient-controlled analgesia pump. Post-operative bleeding occurred in two patients, one patient was re-operated without removing the catheters and the other patient received an intravascular embolization. Local infection was encountered in two patients (3.3%) and was treated with antibiotics, one patient developed a small abscess which was drained after removal of the catheters. Regarding grade ≥ 3 late toxicities, dysphagia (10%) and dryness of mouth (13.3%) were the most commonly reported toxicities, and they were sequelae of the previous EBRT. Osteonecrosis of the mandible occurred in one patient with a tumor recurrence in the floor of mouth, where two catheters had to be placed directly on the bone due to the presence of residual tumor. The patient received partial mandibulectomy and fibula reconstruction. Another patient developed a small area of soft-tissue necrosis (1 cm) in the base of the tongue which was mistaken radiologically for a tumor recurrence and was resected without further treatment. Collectively, there were 28 events of grade ≥ 3 late toxicities recorded in 21 patients (35%).

## 4. Discussion

The available treatment modalities for patients with recurrent head and neck cancers are usually limited. During the initial treatment, most patients undergo surgery, radiation treatment, chemotherapy, immunotherapies, or a combination of these treatment modalities. Since radical curative surgery is not possible in nearly half of these patients, radiation treatment emerges as the alternative local treatment [1,8,30]. The use of brachytherapy with or without surgery in the salvage management of such patients has been previously studied [30,31,32,33,34,35,36,37]. Without surgery, brachytherapy yielded local control and OS rates ranging from 27.5–92.5% and 18.2–43% at 2 years, respectively [30,31,32,33,34,35]. However, other studies have compared results of brachytherapy with and without surgery. Combining both treatment modalities resulted in improved LC and OS rates. Narayana et al. found that patients who were treated with surgical resection and HDR-IRT had an improved 2-year LC compared to the patients who were treated with HDR-IRT alone (88% versus 40%) [36]. Rudzianskas et al. reported an improvement in both 2-year LC (77% versus 47%) and 2-year OS (62% versus 35%) with combined management versus HDR-IRT alone [38].

In the current report, the RFS was 88.1% and 37.3%, and the OS was 39.2% and 16.6% at 3 years and 5 years, respectively. In a systematic review by Rodin et al., 30 studies used brachytherapy in the treatment of recurrent head and neck cancers with and without surgery; 1515 patients were analyzed. More than 95% of the patients were previously irradiated; HDR-IRT was used in 1003 patients. There was no statistically significant difference in the locoregional recurrence rates between both groups. In that report, the included studies were extremely heterogenous regarding the number of patients, the treatment-era (2D versus 3D), the used brachytherapy technique, the reported outcome, and most importantly the applied dose. Moreover, the missing recurrence rates were extrapolated based on the local and locoregional control rates. All these limitations may have led to insignificant results despite the large number of included patients [30].

There is no standard HDR-IRT dose in the recurrence situation, particularly after previous EBRT. In a systematic review including 7 studies by Tagliaferri et al., the most commonly prescribed HDR-IRT doses ranged between 24–40 Gy in 8–10 fractions with most patients being previously irradiated during the primary treatment [38]. The reported median prescribed dose in the current study (30 Gy) is comparable to what has been reported by Tagliaferri et al.

Treatment-related toxicities of salvage surgery and HDR-IRT are considerable. In a prospective study from Spain, sixty-three patients with recurrent head and neck cancers were treated with surgery and HDR-IRT. More than 50% of the patients had grade 3 or greater adverse events, including 3 fatal events [39]. The perioperative mortality rate after surgery for recurrent head and neck cancers 5.2% on average [8]. Severe late complications that can cause substantial limitation in patients’ quality of life include necrosis, dysphagia, or development of fistulas [30]. Kasperts et al. reviewed 704 patients from nine low-dose-rate (LDR) brachytherapy studies. Soft tissue necrosis occurred in 17% of all patients, osteoradionecrosis in 3%, fistulas in 2%, and in 2% sever hemorrhage or carotid blowouts were reported [40].

The reported rates of severe late toxicities after HDR-IRT are variable among published studies, ranging between 5% and 50% [38]. Glatzel et al. reported a mere 6.7% rate of late toxicities grade III and IV using the score of radiation treatment oncology group (RTOG) among 90 treated patients [34]. However, Ritter et al. reported significant late complications in 5.6–22.2% of 94 patients treated with debulking surgery and HDR-IRT with or without concurrent cetuximab-paclitaxel [41]. Other studies reported an overall complication rate as high as 61% [42]. In our study, no perioperative fatalities were reported. We have reported a 35% rate of grade ≥ 3 late toxicities. Nonetheless, we evaluated more toxicities such as dryness of mouth, loss of smell, and loss of taste, which were not reported in similar studies that used HDR-IRT [38]. Furthermore, the large discrepancy in the reported rates of toxicities in similar studies may be attributed to the lack of a standardized reporting of toxicities regarding the used score and the types of examined toxicities. That may have led to over or under estimation of the treatment-related toxicities.

The addition of systemic treatment to surgery and HDR-IRT was examined in one study by Ritter et al. [41]. In a matched pair analysis, two subgroups were compared: surgery and HDR-IRT versus same treatment plus perioperative cetuximab-paclitaxel protocol. Adding systemic treatment improved survival rates without an evident increase in treatment-induced acute or late toxicities greater than grade III [41].

## 5. Conclusions

Salvage HDR-IRT combined with surgery offers a second-line curative treatment option for regionally relapsed head and neck cancers with acceptable outcomes and toxicities. Further multicenter standardized studies are needed to minimize the heterogeneity of patients, tumor, and treatment characteristics, as well as to standardize the reporting of treatment-outcomes and treatment-related toxicities.

## Figures and Tables

**Table 1:** Patient and disease characteristics.

Characteristics	Value (Range; %)
Median age at salvage treatment (range)	65.6 years (15.4–92.7)
Gender	
Males	46 (76.7%)
Females	14 (23.3%)
Primary site	
Oral cavity	14 (23.3%)
Oropharynx	15 (25%)
Hypopharynx	3 (5%)
Sinonasal/Orbit	8 (13.3%)
Nasopharynx	2 (3.3%)
Lymph nodes	14 (23.3%)
Ear	2 (3.3%)
Recurrence Site	
Local recurrence	41 (68.3%)
Lymph nodes	14(23.3%)
Secondary site	5 (8.3%)
Initial T stage	
T0	9 (15%)
T1	12 (20%)
T2	12 (20%)
T3	11 (18.3%)
T4	14 (23.3%)
Tx	2 (3.3%)
Initial lymph-node status	
Positive	30 (50%)
Negative	30 (50%)
Prior neck dissection	
Yes	20 (33.3%)
No	40 (66.7%)
Prior Chemotherapy	
Yes	27 (45%)
No	33 (55%)
Prior EBRT	
Yes	42 (70%)
No	18 (30%)
Median dose of prior EBRT * (range)	60 Gy (32–70)
Salvage surgery	
Radical excision	51 (85%)
Organ-preservation	9 (15%)
Pathology	
Squamous cell carcinoma	54 (90.0%)
Adenocarcinoma	5 (8.3%)
Other	1 (1.7%)
Grade	
Grade 1	2 (3.3%)
Grade 2	38 (63.3%)
Grade 3	18 (30%)
Unknown	2 (3.3%)
LVSI */PNI *	
Yes	44 (73.3%)
No	16 (26.7%)
Margin status	
Negative	19 (31.7%)
Close margin	4 (5%)
Positive margin	11 (18.3%)
Gross residual (debulking surgery)	27 (45%)

* EBRT: external beam radiation treatment; LVSI: lymph-vascular space invasion; PNI: perineural infiltration.

**Table 2:** Details of salvage irradiation.

Variable	Value (%; Range)
Techniuqe	
HDR-IRT *	53 (88.3%)
HDR-IRT + EBRT *	7 (11.7%)
Median HDR-IRT dose	30 Gy (12–40)
Median HDR-IRT dose/fraction	3 Gy (2.5–5)
Median EBRT dose	30 Gy (30–50)

* HDR-IRT: high-dose-rate interventional radiotherapy; EBRT: external beam radiation treatment.

**Table 3:** Distribution of acute and late toxicities.

Acute Toxicities		Late Toxicities	
	No. of Patients (%)		No. of Patients (%)
Pain		Pain	
Grade 1–2	15 (25%)	Grade 1–2	11 (18.3%)
Grade 3–4	10 (16.7%)	Grade 3–4	5 (8.3%)
Mucositis		Mucositis	
Grade 1–2	13 (21.7%)	Grade 1–2	6 (10%)
Grade 3–4	1 (1.7%)	Grade 3–4	3 (5%)
Dysphagia		Dysphagia	
Grade 1–2	8 (13.3%)	Grade 1–2	10 (16.7%)
Grade 3–4	12 (20%)	Grade 3–4	6 (10%)
Dryness of mouth		Dryness of mouth	
Grade 1–2	9 (15%)	Grade 1–2	19 (31.7%)
Grade 3–4	6 (10%)	Grade 3–4	8 (13.3%)
Loss of smell		Loss of smell	
Grade 1–2	2 (3.3%)	Grade 1–2	2 (3.3%)
Grade 3–4	2 (3.3%)	Grade 3–4	3 (5%)
Loss of taste		Loss of taste	
Grade 1–2	5 (8.3%)	Grade 1–2	9 (15%)
Grade 3–4	1 (1.7%)	Grade 3–4	2 (3.3%)
Bleeding	2 (3.3%)	Osteonecrosis	1 (1.7%)
Respiratory infection	2 (3.3%)	Soft tissue necrosis	1 (1.7%)
Local infection	2 (3.3%)		

## Data Availability

Data is unavailable due to institutional restrictions.
